# A107 ANALYSIS OF THE EFFICACY IN TRANSITIONING FROM FOBT TO FIT FOR COLORECTAL CANCER SCREENING AT A SINGLE CENTRE IN ONTARIO

**DOI:** 10.1093/jcag/gwad061.107

**Published:** 2024-02-14

**Authors:** A Nguyen, G Porwal

**Affiliations:** Cambridge Memorial Hospital, Cambridge, ON, Canada; Cambridge Memorial Hospital, Cambridge, ON, Canada

## Abstract

**Background:**

Colonoscopy is the gold standard for detecting colorectal cancer (CRC) and advanced lesions, but is an invasive and carries some risks, with limited availability and accessibility. Alternatively, the fecal occult blood test (FOBT) and fecal immunochemical test (FIT) are both non-invasive, cost-effective screening tests that can also be used to detect CRC, advanced lesions, and polyps, identifying individuals to be prioritized to undergo colonoscopy. FOBT screens for small amounts of blood in stool by detecting heme through a chemical reaction. FIT confirms the presence of blood in stool by using antibodies to detect hemoglobin. Prior to 2019, Ontario employed FOBT as the preferred method for CRC screening in eligible individuals. In early 2019, Ontario transitioned to FIT as the preferred screening test, due its established superior test performance for identifying patients with high risk lesions for colon cancer.

**Aims:**

This single-centre retrospective study analyzed the change in efficacy of detecting advanced lesions, when transitioning from FOBT to FIT, as identified on subsequent colonoscopy

**Methods:**

A retrospective chart review was conducted of approximately 1000 patients undergoing colonoscopy for FOBT or FIT at Cambridge Memorial Hospital, covering the period of transition from FOBT to FIT. Colonoscopies were performed by 10 endoscopists. Patients were stratified into 2 groups based on fecal test type, FOBT (N = 344) and FIT (N = 572). Overall and individual proportions of cancer, polyps, adenomas, advanced adenomas (AA), and sessile serrated adenomas (SSA) detection in the subsequent colonoscopies were calculated for both groups. The efficacy of both tests was then assessed using statistical analysis.

**Results:**

In total, 344 patients were included for FOBT analysis and results included: cancer (5.52%), any polyp (56.69%), adenoma (43.6%), AA (20.64), and SSA (6.1%). In contrast, 572 patients were included for analysis of FIT group and results included: cancer (3.85%), any polyp (83.22%), adenoma (76.92%), AA (43.01), and SSA (12.94%). Cancer detection was similar in the 2 groups. There was significant improvement in polyp, adenoma, advanced adenoma, and sessile serrated adenoma detection with FIT compared to FOBT. This improvement was consistent in all endoscopists, but more pronounced in endoscopists with lower detection rates in FOBT cases.

**Conclusions:**

The use of FIT as a screening stool test, as compared to FOBT, was associated with a significantly improved detection for polyps, adenomas, AA, and SSA, confirming greater accuracy and sensitivity of FIT as a screening tool. This result confirms the premise, at least at a single institution, that by switching to FIT, Ontario has improved colon cancer screening and prevention with more efficient and higher yield utilization of a limited and costly health care resource

**Funding Agencies:**

None

